# The Attachment of Implanted Teeth

**Published:** 1901-09-15

**Authors:** W. C. Barrett

**Affiliations:** Buffalo, N. Y.


### THE ATTACHMENT OF IMPLANTED TEETH.

BY W. C. BARRETT, M.D., D.D.S., BUFFALO, N. Y.

Abstract of paper read before the National Dental Association, Milwaukee, August, 1901.

The attachment of the teeth is by a true gomphosis, the root being inserted into the bony socket, the articulating membrane being the pericementum.

While the teeth for anatomical purposes may be considered as bone, they are really such modifications as make them analagous, but not homologous, to it. The usual conception is that the pericementum of the implanted tooth being destroyed, the union will be analagous to that taking place when the ends of a fractured bone are united. But in the latter instance the sundered or fractured tissues are absolutely homologous—identical in structure. With the implanted tooth there is nothing more than an analogy existing between its cementum and bone.

The pericementum of an implanted tooth being destroyed, the vitality of the cementum, which is dependent upon it for nourishment, must in case of ankylosis be an impossibility. Under such circumstances it is in the highest degree an absurdity to suppose that the ankylosing tissue could become attached to it. The living does not identify itself with the dead, the one functional and the other functionless.

In cases of loss of an implanted tooth, it is always through the formation of osteoclasts, and the resorption of the root succeeds precisely as in cases of normal removal of the deciduous teeth.

In the attachment of replanted teeth there must be a new formation of membrane of periosteal and pericemental nature. Of course the possibility of the revivification of the old dead membrane is too absurd for serious consideration.

The plastic or organizable lymph which is the product of inflammation, may as readily be transformed into membrane, according to the type of that already in existence, as into bone. Admitting, then, what appears most probable, the formation of a new membrane of pericemental nature, what would naturally follow? The deposition of bone upon the periosteal surface through the formation of osteoblast cells, which will unite with the homologous bony walls and fill the existing cavity. There is no instance, so far as we know, in which there has been any new cemental growth in such cases. The new formation must be confined to the osseous tissue, and the new pericementum must closely invest the root. But the fibers of Sharpey may penetrate the cemental structure as they do the bone, and thus hold the tooth as firmly as though it had never been extracted.

### DISCUSSION.

Dr. Fillebrown, of Boston: The suggestions of the essayist are somewhat imaginative rather than veritable. But still theorizing has its use, and it is a question if it be not sometimes more satisfactory to speculate than to search out the hidden things, so far as culture goes. A dentist who confines himself to the routine of mechanical or technical practice never gets a very broad view of his sphere.

In my judgment pyorrhea alveolaris never occurs without local irritation, and its development is dependent on the conditions of the mouth and the structures involved. In a sterile mouth its progress will be slow. In a filthy mouth, and with tissues peculiarly susceptible to inflammation, its progress will be rapid and very destructive. The essayist has not clearly defined the nature of the repair tissue about implanted teeth, and no one has, and so we can only speculate as to its nature and vitality. From clinical experience the testimony goes to show that this tissue does not have an ideal function and that it is liable to the destructive influences of disease.

Dr. Jas. Truman, of Philadelphia: I object to the statement of the essayist that histologically bone and cementum are not homologous. These hard tissues are similar in structure and developed in the same manner, one in smaller quantity and a denser matrix than the other, but still in much the same manner.

I do not believe that pyorrhea is a constitutional disease, but that it is a local disease of microbic origin and susceptible of local treatment. I would divide it into two classes, the adult and the senile, and its treatment will necessarily be based upon which of these classes it belongs to. It is, when at all curable, amenable to local treatment.

Dr. S. H. Guilford, of Philadelphia: I have not scientifically investigated the subject of implanted teeth, but from reading I have come to the conclusion that when a tooth is implanted with the natural pericemental tissue attached, the tooth becomes invested or attached much the same as other cavities in bones are filled in by sponge grafting. The new growth of bone fills in the matrix made by the old membrane. Implanted teeth are often lost because the root is absorbed by the surrounding absorbent cells rather than being reinforced by a deposit of new bone. Just why it should absorb the root in one case, and encyst it in another, we do not know.

Dr. G. V. Black, of Chicago: In the higher animals we never find bone and cementum uniting. When cemental tissue has suffered injury or taken on repair, we always find that it is characteristically cemental tissue. It may not be normal structure, but cicatricial, and yet it is cemental in structure.

Dr. C. N. Pierce, of Philadelphia: Some years ago I experimented in implanting teeth by cutting a groove or slot in the root to afford more chance for mechanical attachment; but I found that teeth treated in this way loosened from absorption of the root much sooner than when the roots were left in their natural condition.

Dr. Hungerford, of Kansas City: Periosteum always develops bone, and pericementum can develop nothing else than cemental tissue. If in extracting a tooth the periosteum is torn out of the socket, the wound will not readily heal. And when the pericemental membrane is dead or detached, no new cement tissue can possibly be formed. So that, when a tooth is implanted in an artificial socket, it never becomes attached in the normal way, but is simply encysted. If there has been no infection there will be no suppuration, and the tooth will become thoroughly encysted.

Dr. E. Noyes, of Chicago, related a case where a tooth was accidentally removed from his wife's mouth, and which was immediately replanted, and it gave good service for thirty years.

Dr. Bogue, of New York City, related a case where two bicuspids were extracted and immediately replanted. Both became firmly reunited and remained so for many years.

DR. J. S. MARSHALL, of San Francisco, related a case of extracting accidentally a bicuspid tooth, about fifteen years ago, which he replanted immediately, and the pulp never died and is in good condition to-day.

